# The Roles of p53 in Mitochondrial Dynamics and Cancer Metabolism: The Pendulum between Survival and Death in Breast Cancer?

**DOI:** 10.3390/cancers10060189

**Published:** 2018-06-08

**Authors:** David E. Moulder, Diana Hatoum, Enoch Tay, Yiguang Lin, Eileen M. McGowan

**Affiliations:** 1 School of Life Sciences, University of Technology Sydney, 15 Broadway, Ultimo NSW 2007, Australia; d.moulder@garvan.org.au (D.E.M.); diana.hatoum@student.uts.edu.au (D.H.); 2 Viral Hepatitis Pathogenesis Group, The Westmead Institute for Medical Research, University of Sydney, 176 Hawkesbury Road, Westmead NSW 2145, Australia; enoch.tay@sydney.edu.au; 3 Central Laboratory, The First Affiliated Hospital of Guangdong Pharmaceutical University, Guangzhou 510080, China

**Keywords:** breast cancer, p53, tumor suppressor protein, p14ARF, metabolism, mitochondria

## Abstract

Cancer research has been heavily geared towards genomic events in the development and progression of cancer. In contrast, metabolic regulation, such as aberrant metabolism in cancer, is poorly understood. Alteration in cellular metabolism was once regarded simply as a consequence of cancer rather than as playing a primary role in cancer promotion and maintenance. Resurgence of cancer metabolism research has identified critical metabolic reprogramming events within biosynthetic and bioenergetic pathways needed to fulfill the requirements of cancer cell growth and maintenance. The tumor suppressor protein p53 is emerging as a key regulator of metabolic processes and metabolic reprogramming in cancer cells—balancing the pendulum between cell death and survival. This review provides an overview of the classical and emerging non-classical tumor suppressor roles of p53 in regulating mitochondrial dynamics: mitochondrial engagement in cell death processes in the prevention of cancer. On the other hand, we discuss p53 as a key metabolic switch in cellular function and survival. The focus is then on the conceivable roles of p53 in breast cancer metabolism. Understanding the metabolic functions of p53 within breast cancer metabolism will, in due course, reveal critical metabolic hotspots that cancers advantageously re-engineer for sustenance. Illustration of these events will pave the way for finding novel therapeutics that target cancer metabolism and serve to overcome the breast cancer burden.

## 1. Introduction

Nicknamed the “Guardian of the Genome,” p53 (encoded by the TP53 gene) is believably the most extensively studied and most multifaceted tumor suppressor protein identified to date [1,2,3]. The role of p53 extends well beyond its role as a tumor suppressor; it is emerging as an important regulator of metabolic homeostasis, pivotal in most major cellular processes [1,4,5,6,7,8]. In the breast p53 plays a fundamental role in cell homeostasis: maintaining metabolic homeostasis during pregnancy, providing a protective role against latent breast cancer [9], and its more classical roles of protecting the genome, DNA repair, and/or programmed cell death [10]. Critical mutations in the TP53 gene are common in most cancers and major contributors to cancer progression. In contrast, the majority of breast cancers harbor wild-type p53 (wt-p53), though in most cases the protein is non-functional, in part due to loss of upstream regulators in the p53 pathway [11]. Classical thinking supports that reinstating the p53 pathway may be one avenue of more efficacious breast cancer treatment through p53 activation of programmed cell death pathways. This concept may be flawed due to the poorly understood role of p53 in cellular metabolism, which is currently being widely revisited.

Originally, altered metabolism was deemed to be merely a byproduct of cancer, rather than being considered as playing an intricate role in its support and maintenance. In the last decade, however, the resurgence of cancer metabolism research has increased exponentially. Cancer metabolism is now being viewed as a series of critical metabolic reprogramming events within biosynthetic and bioenergetic pathways, needed to fulfil the requirements of cancer cell growth and maintenance [1,12,13,14]. Renewed focus on cancer metabolism has identified the tumor suppressor protein, p53, as a key regulator of metabolic processes and metabolic programming [1,15,16,17].

This review firstly provides a brief understanding of ongoing problems with breast cancer therapy. Next, we provide an overview of the classical and non-classical roles of p53 focusing in on the emerging, critical roles of p53 in metabolic reprogramming. On one hand, p53 engages in mitochondrial cell death processes in the prevention and treatment of cancer, and on the other hand it plays an important role in cell survival and function, hence ‘balancing the pendulum between cell death and survival’. Further, we bring back the discussion to how these p53-reprogramming events may be important in cell survival and provide new avenues for breast cancer therapies.

### 1.1. Overview of Breast Cancer

Breast cancer is the most commonly diagnosed cancer and cause of cancer deaths among women worldwide: an estimated 1.7 million cases were diagnosed in 2014, accounting for 25% of all cancers in women [18]. One in eight women will develop breast cancer in their lifetime [19,20]. Upon diagnosis, breast cancers are categorized into subtypes that are defined based on their stage of progression, level of invasiveness, and hormonal receptor status [21,22,23]. There are three distinct classes of hormone receptor profiles that are typically overexpressed and highly dysregulated in breast cancer. These include: (1) the estrogen receptor (ERα)/progesterone receptor (PR); (2) human epidermal growth factor receptor 2 (HER2); and (3) triple-negative breast cancers (TNBC) that do not express any of these three receptors [21,22,23]. The prevalence of each of these subclasses and their typical treatments are summarized in Table 1. Common treatment regimes for breast cancer include surgical ablation, adjuvant or neoadjuvant chemotherapy, radiotherapy, and endocrine therapy, with the therapeutic strategy dependent on the stage and type of cancer [24]. Radiotherapy and chemotherapy induce DNA damage, inducing cell cycle arrest and apoptosis [25,26,27,28]. Endocrine therapy, including Tamoxifen and Fulvestrant, blocks the ERα receptor, which in turn represses aberrant estrogen signaling [21]. Similarly, targeted therapies like Trastuzumab target the overexpression of HER2 [21,29], while aromatase inhibitors inhibit the synthesis of estrogen [21,30].

### 1.2. Breast Cancer Treatment Resistance and Recurrence

Current therapeutics, used alone or in combination, have been successful in the treatment of breast cancer, with favorable increases in overall survival in recent decades [33]. Unfortunately, a major challenge involves a large proportion of breast cancer patients developing resistance to these treatments (50% of ERα+/PR+ breast cancer, which comprises 70–80% of all breast cancers, inevitably recur) [21,34,35]. Resistance leads to the recurrence of more aggressive secondary tumors [36,37], stressing the need for further research into the fundamental cellular mechanisms that promote breast cancer tumorigenesis, resistance, and recurrence. More recently, targeting dysregulated metabolism is a reemerging area of cancer research. Metabolic reprogramming is now being recognized as a cancer hallmark. 

To date, breast cancer research has been heavily focused on exploring the contributions of hyperactive growth and hormone receptor signaling, inherited and sporadic gene mutations, oncogene amplification, loss of tumor suppressor proteins, and cancer heterogeneity in the development and progression of breast cancer. For instance, mutations in the breast cancer genes BRCA1 and BRCA2 as well as the TP53 gene have all been well characterized in breast cancer [38,39,40,41]. Aberrations in the P13K/AKT signaling pathway, which regulates the cell cycle and cell proliferation, are also well documented [42]. In contrast, aberrant metabolism, as a contributing factor to the onset, progression, and maintenance of breast cancer, is poorly understood. 

In contrast to most cancers, mutations in the TP53 gene are only present in 20–30% of all breast cancers, suggesting that p53 function is compromised [43,44] (discussed in Section 4). Alongside its well-recognized role as a tumor suppressor, p53 has emerged as a major regulator of metabolic processes and metabolic programming and is intrinsically linked with mitochondria and mitochondrial dynamics associated with cell survival [1,15,16,17,45]. Further, more and more evidence is emerging to suggest an association between drug resistance and dysregulated cellular metabolism [46]. In the normal breast, during pregnancy p53 plays a key role in the reprogramming of breast cell function and is strongly associated with protection against latent breast cancer. However, it is also associated with the seeding of latent breast cancer, as reviewed in [9]. 

What key knowledge are we missing regarding the role p53 plays as the ‘guardian of metabolism’ that will help us understand breast cancer development, survival, resistance to treatment, and impact on breast cancer recurrence?

## 2. The Multifaceted Tumor Suppressor Roles of p53

The p53 protein is a transcriptional regulator that is stabilized within the nucleus upon DNA damage or oncogenic signaling. p53 regulates genes involved in cell cycle arrest, cell death (apoptosis), DNA repair, and senescence to prevent tumor development and growth [3,5,47,48,49,50,51]. p53 also localizes to the cytoplasm and the mitochondria, where it can regulate cytoplasmic cellular functions including apoptosis. Non-canonical functions of p53, including necrosis [52], autophagy [53], and the less well-known functions of p53, necroptosis (inflammatory programmed cell death) [54] and ferroptosis (iron- and lipid-mediated cell death), have all been shown to be p53-activated specialized forms of mitochondria-programmed cell death processes [5,50,51]. Critical mutations in p53 can prevent its anti-tumor functions and/or enhance oncogenicity [55,56]. All these p53 functions, which are briefly discussed as part of this review, are clearly directed towards the physiology of removing abnormal cells and prevention of tumorigenesis. Additional less characterized p53-associated processes that do not necessarily lead to cell death include autophagy, senescence, differentiation, and dormancy. In recent years, mounting evidence has also implicated p53 as a central player in the regulation of cellular energy metabolism [1,10,15,16,17], alongside potential roles in the regulation of mitochondrial dynamics, beyond cell death [57,58,59]. Homeostasis regulation of the mitochondrial DNA involves both p53-nuclear transcriptional target genes, whose products translocate to the mitochondria or non-nuclear direct cytoplasmic effects of the p53 protein.

### 2.1. Canonical and Non-Canonical Functions of p53 in Cancer Protection

**Figure 1:**
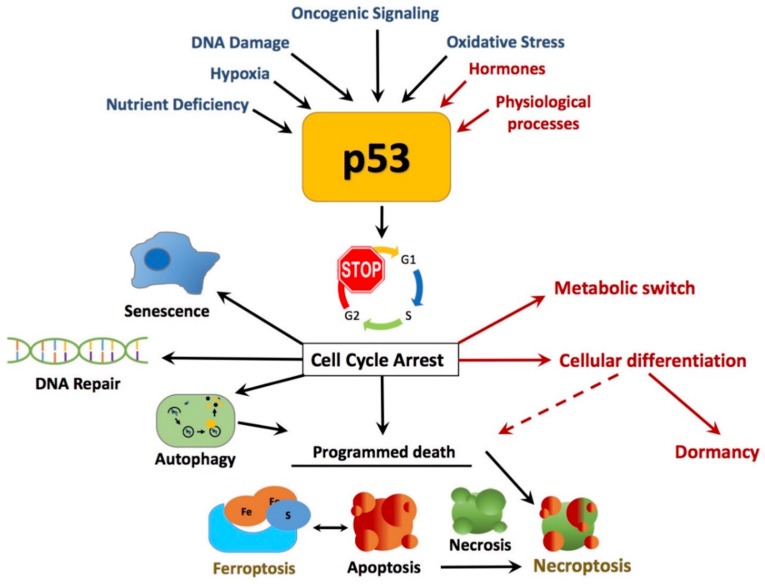
p53 canonical and non-canonical tumor suppressor roles of p53. p53 is activated by a range of cellular stress signals. These activators of p53 include nutrient stress, hypoxic conditions, activation of oncogenes, DNA damage, and oxidative stress from reactive oxygen species (ROS) and, as a result, increase the activity of p53. Classical or canonical responses of p53 include, transcriptionally and translationally, cell cycle arrest and repair damage to DNA, which place the cell in a state of senescence or induce apoptosis. Non-canonical, controlled programmed cell death roles include autophagy pathways, necrosis, necroptosis, and ferroptosis [5,48,50,51,52,53,62,63,64]. Normal physiological processes such as hormone activation can also lead to p53-induced cell cycle arrest and p53 acts as a switch in metabolic process involved in differentiation, redirecting specialized cell function [9].

Much of the p53 literature is directed towards the well-characterized canonical tumor suppressor protective roles of p53, which are typically impaired upon mutation or deletion, leading to the production of many of its oncogenic forms [15,39,60]. Oncogenic p53 mutations, resulting in change or loss of function, are found in approximately 50% of all human cancers, making it one of the most frequently mutated proteins in cancer [39,61]. From its widespread transcriptional factor activity, p53 has the ability to activate the expression of genes associated with cell cycle arrest, cell death, DNA repair, and cell abeyance, which occurs in response to a range of cellular stress signals that post-translationally activate p53 (Figure 1) [48,53,62,63].

#### 2.1.1. DNA Damage

One of the early identified major functions of p53 is to prevent accumulation of damaged DNA to potentiate genomic stability, thus inhibiting tumorigenesis [65]. This is achieved by temporarily inhibiting cell proliferation to allow for the repair of DNA damage prior to DNA synthesis, or, in the case of irreparable DNA damage, initiate programmed cell death to remove mutated cells. The ability of p53 to initiate DNA repair is conventionally through stimulating the transcription of the downstream cell cycle inhibitor p21 and hence triggering DNA damage repair mechanisms [66,67,68,69]. Unsurprisingly, the p21 regulatory checkpoint machinery is commonly overridden in cancer [68]. In response to cell cycle arrest, DNA repair mechanisms are frequently upregulated to repair the respective DNA damage before the cell is permitted to re-enter the cell cycle. Specifically, these include the confirmed roles of p53 in transcriptionally coordinating genes, allied to the nucleotide excision and base excision repair pathways [68,70,71]. These repair pathways mediated by p53 carry out DNA repair functions including single-stranded break repair and the repair of other DNA lesions within the genome to prevent genomic instability. Paradoxically, it is mutations in the p53 gene itself that lead to tumor formation.

#### 2.1.2. Apoptosis

One of the most well-recognized canonical p53 programmed cell death pathways is apoptosis. Nuclear p53 regulates genes associated with apoptosis in response to cellular stress signals via the classical mitochondrial apoptotic pathway [49]. The primary mechanism by which p53 induces apoptosis is transcriptional regulation of the pro-apoptotic proteins NOXA and p53-upegulated modulator of apoptosis [72,73,74]. An additional mechanism by which p53 induces apoptosis is direct translocation to the mitochondria, activating the mitochondrial apoptotic regulator BAX [75]. This implies that p53 mediates apoptosis both by transcriptional and post-translational mechanisms. However, the question of how p53 activation induces apoptosis outside of its other well-known cellular fate functions remains unclear. One proposal highlights that p53 may need to reach an “apoptotic threshold” that overrides other functions to correspondingly promote apoptosis [49]. Other studies suggest that the strength, duration of activation, sub-cellular location, and environmental co-factors of p53 play key roles in determining the downstream outcomes of p53 activation [48,49,76]. 

#### 2.1.3. Necrosis

Necrosis was once regarded as uncontrolled or ‘accidental’ cell death. This idea has been superseded; necrosis is now regarded as a process of irreversible programmed cell death where apoptosis has failed and can also be triggered by p53 [52,64,77]. Necrosis is a caspase-independent pathway requiring receptor interaction protein kinases 1 and 3 (RIP1 and 3) and can be mediated by p53 [54,78]. Subsequently, this action directly or indirectly affects mitochondria through NADPH oxidase-derived ROS. The terms of necrosis have extended to include programmed necrosis, regulated necrosis and necroptosis. *Necroptosis* is a relatively new concept, which is a combination of necrosis and apoptosis-mediated p53 cell death and contributes to immune system regulation, contributing to managing cells during inflammation, tissue injury, and other organismal stresses such as pathogen infection [78,79].

#### 2.1.4. Autophagy

The definition of autophagy, from an ancient Greek word, is ‘self-devouring’. Autophagy is an evolutionarily conserved, genetically mapped process that forms the “recycling facility” of the cell. This is a normal process in the cell to maintain homeostasis through programmed protein degradation and turnover of organelles within the cell. This allows the cell to degrade organelles and proteins to functional building units to fuel bioenergetic and biosynthetic processes [80]; alternatively, cancer cells can undergo autophagy as a mechanism of cell death [81]. This catabolic pathway, involved in both normal cell physiology and cancer pathophysiology, is modulated by p53-dependent mechanisms, which is dependent on p53 cellular localization [82,83,84,85]. Within this context, p53 is seen to have a dual/paradoxical role in regulating autophagy. On the one hand, the nuclear p53 function has been shown to transcriptionally activate pro-autophagy genes; conversely, its cytoplasmic role shows evidence of negatively regulating autophagy [82,83,85]. Therefore, the cellular context and environment determines the role of p53 in autophagy signaling pathways. Autophagy is also important in selective degradation of mitochondria (mitophagy) in mitochondria quality control [86]. In cancer, autophagy has a controversial role, and is associated with interventions to both stimulate and inhibit cancer, as debated in the recent review by Thorburn [87].

#### 2.1.5. Dormancy

Perhaps one of the most under-researched roles of p53 is dormancy. It has been suggested that dormancy may be viewed as a natural byproduct of evolutionary mechanisms, where dormant cells are present in both healthy individuals and survivors of cancer [88]. The dormant cell minimizes energy expenditure to survive in adverse conditions. Mitochondria undergo active to deactivate transition; however, the exact mechanism of this transition is unclear [89]. In breast cancer the concept of cancer dormancy is particularly relevant given that most breast cancers are seeded early in life and yet only manifest in later years. Many years after seemingly successful breast cancer treatment, 20–50% of breast cancers recur, believed to reemerge from remnant dormant cells from the original tumor [90,91]. How this is achieved is unknown. Autophagy has been suggested as one of the key processes to long-term cancer survival, allowing stressed cells to remain dormant yet viable in order to survive and eventually regrow and relapse [91,92]. An additional theory is that tumor dormancy results from tumor cells reaching a differentiated state [91]. In our laboratory we have shown that p53 can induce a dormant state in breast cancer cells, which has features of differentiation or senescent-like morphology [59,93,94]. We also demonstrated that these breast cancer cells can remain viable for many weeks in cell culture after p53-induced cell cycle arrest and eventually multiply by a process of endoreplication or nuclear replication without mitosis [93]. Endoreplication may be one method by which breast cancer cells escape dormancy and become more aggressive.

#### 2.1.6. Cellular Senescence or Quiescence or Dormancy

p53 is a decision maker in cellular senescence, quiescence, or dormancy. Senescence is defined as a state of irreversible cell cycle arrest in which cells display an inability to proliferate or respond to growth factors [95,96,97,98]. In vivo studies highlight the pivotal role that p53-induced senescence plays in tumor suppression [95,98]. Quiescence, a prolonged cell cycle arrest and attenuated cellular activity at the G0–G1 phase, is also mediated by p53 [99,100]. Alternatively, dormancy is not unheard of in terms of breast cancer recurrence, with 20–50% of all breast cancers recurring from years to decades post treatment [35,90]. A common attempt to explain dormancy arises from models of both senescence and quiescence, both mediated by p53. However, understanding the paradox of how these potentially overlapping, and supposedly anti-tumorigenic functions facilitate dormancy and lead to recurrence remains incomplete [91]. One potentially unidentified area to be investigated involves the effects of p53 within cancer metabolism throughout and leading up to the point of recurrence. 

#### 2.1.7. Ferroptosis

Only discovered in 2012, ferroptosis is a novel non-apoptotic form of cell death, characterized by lethal iron-dependent accumulation of lipid ROS, in a caspase-independent manner [101]. Morphologically, cells undergoing ferroptosis cell death are distinguished by small mitochondria with condensed membrane density. Dixon and colleagues discovered that ferroptosis action can be blocked or reversed by ferrostatin (Fer-1) in cancer cells [101]. Fer-1 has been shown to reverse ferroptosis action through increasing the mitochondrial membrane potential and decreasing ROS accumulation [102]. To investigate the process of ferroptosis, Gu and colleagues generated a mouse model (named p53) that was unable to undergo p53-dependent cell cycle arrest, apoptosis and senescence, but was still able to regulate p53-dependent changes in energy metabolism and ROS production [103]. This suggested that the p53 associated energy metabolism and ROS functions were disconnected from conventional cell cycle arrest and death mechanisms. Specific p53 target genes have been identified as important in p53-directed ferroptosis including solute carrier family 7 member 11, a cysteine-glutamate exchanger, glutamase 2 (GLS2), prostaglandin-endoperoxide synthase 2, and spermidine/spermine N1-acetytransferase1 [5]. This unique form of cell death is implicated in multiple disease states including cancer [50]. 

In summary, mitochondria are core powerhouses for metabolic reactions that drive cellular reprogramming through diverse pathways and mechanisms. Classical roads in p53 tumor suppression lead to mitochondrial de-regulation or inactivity, whether it be for cell destruction or temporary/permanent inactivation, respectively. Alternatively, p53 is linked to the quality control mechanisms of the mitochondria and its genome, with projected roles in the organelle fusion–fission process (discussed in Section 3.4) and supporting cell survival. 

## 3. Emerging Roles of p53 in Cancer Metabolism

Alteration in cancer cell metabolism is now considered a hallmark of cancer, whereby the metabolic signatures of cancer cells are distinctly different from normal tissue [12,14,104,105,106]. These observed differences reflect metabolic changes that strategically fuel the requirements for tumor development. p53 has emerging roles in counteracting the changes that occur in the metabolism of cancer cells through regulation of several metabolic targets [1,10,15,16,17].

### 3.1. Metabolic Reprogramming for Cellular Proliferation

Cellular division requires an adequate biomass to produce two identical daughter cells. This growth imposes a metabolic burden, requiring the cell to increase its energy supply to meet the requirements for proliferation [13,107]. In cancer, the normal regulatory checkpoints within cellular division are lost, permitting the cell to unceasingly proliferate [108]. Re-engineering of fundamental metabolic pathways and nutrient uptake mechanisms often occurs to support this uncontrolled proliferation. This ‘metabolic switch’ or metabolic reprogramming provides inherent growth advantages in fulfilling the requirements of cancer cells [12,13,14,104]. These alterations include: (1) restructuring the key metabolic pathways involved in glucose catabolism; (2) maintaining the nutrient uptake mechanisms to meet metabolic demands; and (3) quickly replenishing anabolic substrates needed for biosynthetic pathways (anaplerosis) such as nucleotide, protein, and lipid synthesis [12,13,14,104]. These observations stem from a fundamental metabolic switch, formally known as the Warburg effect (described in Section 3.2), whereby cancer cells show high dependence on glycolysis and attenuated mitochondrial respiration [106,109,110]. To provide this demand for glucose, cancer cells typically overexpress glucose transporters, supporting glucose flux [111,112]. This reliance on glycolysis, however, is not sufficient for cellular replication. Instead, principal anabolic substrates found within and entering the tricarboxylic acid (TCA) cycle serve as hybrid intermediates, forming the building blocks needed for proliferation [113]. One of these includes the reliance of cancer cells on glutamine, which is converted to α-ketoglutarate within the TCA cycle [113]. Furthermore, cancer proliferation requires increased fatty acid synthesis for membranes and lipid molecules. As such, controlling fatty acid synthesis has been proposed as a potential therapeutic strategy [114,115]. Collectively, these metabolic strategies are employed to support cancer growth and maintenance and, without such adaptive strategies, cancer cells will likely undergo apoptosis. 

Now considered a hallmark of cancer, aberrant metabolism in cancer is re-emerging as an area of intense research. As such, rectifying this aberrant metabolism is an appealing potential cancer treatment. It is expected that intervention leading to interference with cancer cell metabolism would form a successful treatment strategy. Before the development of a treatment strategy based on metabolism intervention, a deep understanding of how cancer metabolism is reprogrammed and regulated is needed. Recently, the tumor suppressor p53 has been implicated as a master regulator of metabolism [1,10,15,16,17]; therefore, understanding the role of p53 in the regulation of cellular metabolic processes may provide further avenues for the future treatment of cancer. 

### 3.2. The Warburg/Weinhouse Debate

**Figure 2:**
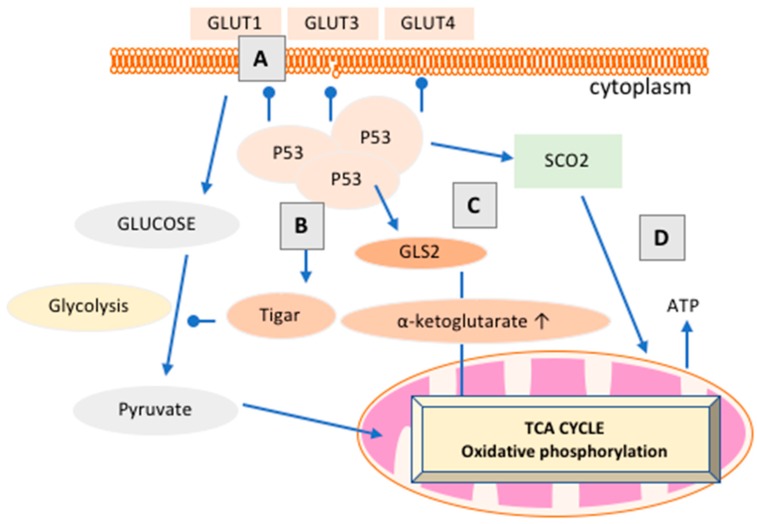
p53 balances glycolysis and mitochondrial respiration. The roles of p53 in cancer metabolism include: (**A**) suppressing the first step of glycolysis by direct downregulation of glucose-type transporters (GLUT) including GLUT 1, GLUT3, and GLUT4 receptors, which are typically overexpressed in the membranes of cancer cells to facilitate glucose flux [110,133]; (**B**) negative regulation of glycolysis by increasing expression of TP53-induced glycolysis regulator (TIGAR) [133]; (**C**) regulation of glutaminase-2, leading to an increase in the metabolite α-ketoglutarate. This, in turn, promotes the TCA cycle and mitochondrial respiration [134]. (**D**) The upregulation of the cytochrome C oxidase (COX) complex, via p53 targeting the cytochrome c oxidase assembly protein, increases mitochondrial respiration. COX is a vital transmembrane protein that accepts oxygen in mitochondrial respiration [17]. This figure has been adapted from [1].

*The Metabolic Switch*. The concept of cancer metabolic switching from oxidative phosphorylation to glycolysis (Figure 2) was first introduced by Otto Warburg in 1930 [106,109,110]. This shift in cellular metabolism to a dominant glycolytic state, termed “The Warburg Effect” (aerobic glycolysis), is unexpected as glycolysis is preferentially utilized over mitochondrial respiration, a mechanism that supplies more energy, irrespective of the presence of oxygen [106,109,110]. 

Since then, the Warburg effect has been extensively studied, with many proposals attempting to reveal both its origins and its promotion of tumorigenesis [106,109,110]. Originally, Warburg proposed that damage to mitochondrial respiration (OXPHOS) in all cancers is sufficient for a malignant glycolytic state, thus tumorigenesis is facilitated [116]. It is argued that the silencing of mitochondrial respiration, leading to the Warburg effect, is dependent on somatic and hereditary mutations in both nuclear and mitochondrial DNA (mtDNA) [109]. Tumor-promoting mutations have been found in mitochondrial encoded genes within prostate cancers and head and neck tumors [117,118]. Furthermore, nuclear DNA mutations in a number of mitochondria-specific metabolic enzymes have been shown to facilitate tumorigenesis, leading to the formation of paragangliomas and pheochromocytomas [119,120]. However, these mutations are considered rare, and are not associated with more commonly occurring cancers such as breast cancer [109]. The Warburg theory has been hotly debated and there is no sound experimental evidence that mitochondrial metabolism is impaired in all cancer cells [121,122,123]. Apart from a limited range of rare cancers, damage to mitochondrial respiration is not the driver of the glycolytic shift; in fact, many cancers exhibiting the Warburg effect have been shown to retain functional mitochondria (normal OXPHOS) [109]. A biochemist, Sidney Weinhouse, a pioneer in metabolic biochemistry, found that the cancer cell was able to oxide glucose and fatty acids at a similar rate to normal cells [124]. One early explanation was that anaerobic glycolysis was so high in tumor cells that it eliminated the need for oxidative respiration [121]. Hence a chicken-and-egg analogy [125]. Did the mitochondrial dysfunction occur first, pushing the cell to preferentially use glycolysis? Alternatively, did an increased flux in glycolysis occur first, thereby repressing oxidative respiration? Neither viewpoint has been shown to be right or wrong, and this conundrum is discussed in detail in the review by Senyilmaz and Teleman [125]. There is some evidence to suggest that the glycolytic phenotype is characteristic of highly proliferating cells, whereas the switch to oxidative respiration occurs during differentiation [126]. This explanation is in line with our research, which shows that induction of p53 blocks the cell cycle and increases both oxidative respiration and mitochondria biogenesis in breast cancer cells [59,93]. 

As damage to mitochondrial respiration does not appear to be the cause of glycolytic metabolic dominance, the question is: What are the crucial drivers in the manifestation of Warburg’s original observations? High mitochondrial activity and a dependence on mitochondrial and glycolysis metabolism have been shown to be essential for the rapid proliferation of tumors [127,128].

### 3.3. How Does p53 Regulate Cancer Metabolism?

A loss of tumor suppressor proteins, oncogenic mutations within glucose metabolism, and changes in the tumor microenvironment serve as the key drivers of metabolic reprogramming of aerobic glycolysis [12,129,130,131,132]. It is only in the last 10–15 years that the link between p53 and aberrant metabolism has been suggested [1,10,15,16,17,107]. As discussed above (Section 3.1 and Section 3.2), enhanced aerobic glycolysis and attenuated mitochondrial respiration facilitate cancer proliferation via energy production and biosynthetic pathways. As summarized in Figure 2, p53 has been shown to downregulate a number of critical components of the glycolytic pathway, including glucose entry into cells [1,4]. The net result of p53 activation appears to be the inhibition of the overproduction of pyruvate, the end product of glycolysis, and the promotion of mitochondrial respiration, as exhibited within normal tissue. Typically, cancers preferentially convert this build-up of pyruvate to lactate, but it can be converted to acetyl-CoA by lactate dehydrogenase and pyruvate dehydrogenase (PDH), respectively [10]. p53 has been shown to negatively regulate mitochondrial PDH 2, which increases PDH activity, thus promoting the conversion of pyruvate to acetyl-CoA instead of lactate, encouraging the TCA cycle, and enhancing mitochondrial respiration [10]. p53 has also been shown to regulate fatty acid oxidation (FAO) by facilitating the transport of fatty acids into the mitochondria via activation of carnitine palmitoyltransferase 1C and by positively regulating the β-oxidation of fatty acids in response to nutrient stress [10]. This ability of p53 to increase FAO promotes NADH and FADH2 production, which enhances OXPHOS [10].

Cancer cells typically increase fatty acid uptake and synthesis to meet the demands of membrane biosynthesis, and p53 has been shown to oppose this effect. In contrast, early literature suggests p53 promotes glycolysis in a tissue- and context-specific manner, where p53 is activated to rectify a metabolic stress [135,136]. These include the positive regulation of enzymes that control the first rate-limiting step of glycolysis, such as hexokinase II (HK2), by p53 [135,136]. More recently, p53 has been shown to play a crucial role in re-aligning metabolic homeostasis to ensure cell survival during nutrient deprivation [137]. These examples shed light on an emerging view of p53 as a metabolic homeostatic regulator in both normal and cancerous tissue, and not solely as a mediator of the glycolysis and OXPHOS metabolic balance [10].

### 3.4. Mitochondrial Dynamics and p53

Sustaining metabolic homeostasis not only relies on the activity of tumor suppressor proteins such as p53, but also on mitochondrial regulation. This includes controlling mitochondrial size, number and shape through fusion and fission events and intracellular transportation [138,139]. These mechanisms, known as mitochondrial dynamics, ensure optimal mitochondrial bioenergetic function to accommodate energy demands of the cell [140,141]. Mitochondrial dynamics has been observed to be influenced by p53 [57,59,142]. 

**Figure 3:**
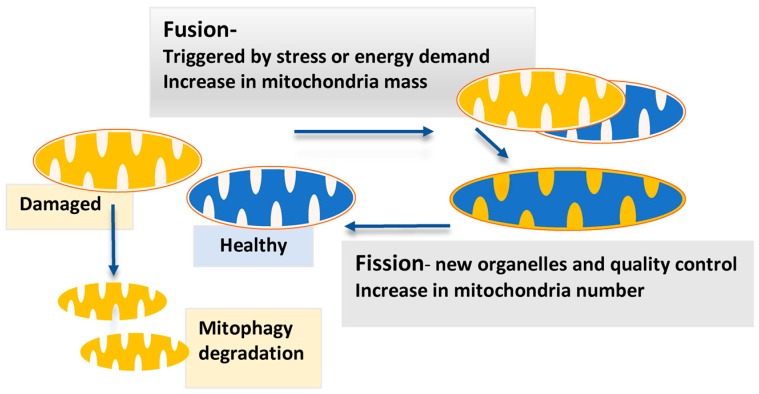
Mitochondrial fission–fusion cycle sustaining mitochondria function, number and genetic health. Under stressful or energy-demanding conditions, mitochondria undergo fusion to complement damaged (yellow) and healthy (blue) mitochondria. This allows for a mixing of constituents alongside increasing membrane surface area, which optimizes bioenergetic functioning. An imbalance between fission and fusion—for instance, greater fission—leads to mitochondrial fragmentation and may increase the number of mitochondria if mitophagy does not eliminate mitochondria. Conversely, more fusion is seen to form large tubular networks. The biogenesis of mitochondria occurs to increase mitochondrial biomass or compensate for mitochondrial degradation. Thus, imbalances between mitochondrial fission, fusion, biogenesis, and degradation appear to regulate the mitochondrial number, shape, size, and biomass [141,144]. Mitochondrial fission has been associated with sensitizing cells to apoptosis during highly stressful conditions and with environments of nutrient excess. However, fission has also been implicated in the “housekeeping” of mitochondria to produce new mitochondria (blue) and remove old/damaged mitochondria.

The morphology of mitochondria is intimately linked to their functional state [138,140,141]. Mitochondrial fission and fusion are critical balancing events that occur to maintain mitochondrial function when cells are exposed to a wide array of metabolic and environmental stresses [140,141,143]. This includes maintaining mitochondria biomass, number, biogenesis, and their degradation (Figure 3). When fission is unopposed, mitochondrial fragmentation occurs, and is associated with excess glucose abundance, severe stress, cellular death, and impaired OXPHOS. However, fission has also been observed to be vital in the generation of new mitochondria (mitochondrial biogenesis), alongside a quality control process to remove old defective mitochondria, ensuring proper mitochondrial function (Figure 3) [140,141]. 

Unopposed mitochondrial fusion is observed in mitigating stress during nutrient withdrawal and enhancing OXPHOS rates. This occurs by removing defective mitochondria via the fusing with functionally healthy mitochondria in a complementation process [140,141] (Figure 3). There is some evidence that mitochondrial fission and fusion are regulated by p53: Mitofusin2 (mfn2), an integral membrane bound component of fusion, has been shown to be a direct downstream target of p53, implicated in stopping cellular proliferation and sensitizing cell death [57,140].

Within muscle physiology studies, p53 has been observed to regulate alterations in fission and fusion proteins, with p53 knockout (KO) mice displaying acute alterations in mitochondrial morphology and reduced respiratory capacity [58]. This coincided with a former study revealing a loss of mitochondrial function and biomass in p53 KO mice, while wild-type p53 mice maintained mitochondrial biogenesis [142]. The influence of p53 in mitochondrial dynamics is yet to be investigated in breast cancer metabolism, alongside the pivotal role of p53 in mediating the balance between glycolysis and OXPHOS, a current focus in our laboratory. This may prove vital as mitochondrial dynamics appear to serve key metabolic roles towards changing metabolic demands and bioenergetic efficiency of the cell. 

### 3.5. p53 Family Members p63 and p73 Play Significant Roles in the Cancer Metabolic Switch

While this review is focused on the functions of the major p53 isoform, some of the smaller alternatively spliced p53 isoforms have been shown to enhance p53 target expression or inhibit p53 wild-type function [145], especially in breast cancer [146]. Two other p53 family members, p63 and p73, also modulate p53 function [4]. Although p53, p63, and p73 isoforms are highly homologues, sharing similar structure and functions as transcriptional factors, studies in KO models of p53, p63, and p73 show functional diversity as well as overlapping functions [147,148,149,150,151]. Interestingly, akin to p53, recent studies show that p63 and p73 isoforms (TAp63, ∆Np63, Tap73, and ∆Np73) partake in glucose metabolism [4]. However, this shared relationship proves to be divided, as p63 and p73 isoforms exhibit effects both similar and opposite to those of p53. Whereas p53 is known to inhibit glycolysis and induce fatty acid oxidation, TAp63-null mice develop associated defects in glucose uptake, leading to insulin resistance, obesity, and glucose intolerance. Moreover, although controversy over p53’s roles in the phosphate pentose pathway (PPP) lingers, TAp73 appears to enhance PPP flux through increasing the expression of glucose-6-phosphate dehydrogenase, therefore supporting cancer proliferation [152]. 

As mentioned, p63 and p73 do exhibit similar effects or share overlapping functions with p53. These include TAp63 and TAp73’s ability to inhibit glycolysis; the function is similar to that of p53, although the mechanisms vary. Both TAp63 and TAp73 have been shown to induce islet amyloid polypeptide to inhibit glycolysis via inhibiting HK2 [153]. The TAp63 isoform, akin to p53, is associated with inducing GLS2, therefore promoting the TCA cycle [154]. TAp73 is associated with supporting mitochondrial function via oxygen consumption and complex IV stability [155]. This aligns nicely with recent evidence of p53’s role in mitochondrial function, as reviewed in [4]. Collectively, p63 and p73 isoforms’ effects on glycolysis are influenced by the cellular environment, although they both seem to promote the TCA cycle and PPP. 

## 4. p53 and Breast Cancer Metabolism

Breast cancer metabolism is diverse and very much dependent on hormone fluctuation [156]. Metabolic rewiring is also determined by breast cancer subtypes [157]. Mutations in p53 have been identified as drivers of aberrance in oxidative respiration and glycolysis in breast cancer, also dependent on the breast cancer sub-type [156,157]. Contrary, most hormone-dependent post-menopausal breast cancers, which make up the major breast cancer sub-type, have wt-p53. p53 plays a complex role in normal breast and breast cancer metabolism, complicated by the constant flux of female hormones, as reviewed in [9]. In breast cancer, p53 has been associated with the modulation of key proteins in mitochondrial metabolism, cytochrome c oxidase 2 synthesis, and the TP53-induced glycolysis and apoptosis regulator (TIGAR) [158]. Targeting p53 in its role as a metabolic switch is an exciting but underexplored area of breast cancer treatment. In fact, drugs that target the p53-dependent metabolic checkpoint such as metformin (a commonly used medication for type 2 diabetes to reduce glucose production) are undergoing clinical trials in combination with established therapies [159]. 

### 4.1. Upstream Regulation and Reactivation of p53 in Breast Cancer

As mentioned in Section 1.2, only 20–30% of breast cancers sustain TP53 mutations, leaving ~70% retaining functional wild-type-p53 (wt-p53) [43,44]. Under homeostatic conditions, cellular p53 protein levels are low due to its short half-life and continual degradation by the HDM2 ubiquitin ligase [160,161]. Due to the large proportion of breast carcinomas retaining wt-p53, radiotherapy and chemotherapy agents such as doxorubicin are successful in reactivating p53 via the DNA damage pathway, inducing cell cycle arrest and cell death [25,26,27]. In line with its classical role as a tumor suppressor, novel anti-HDM2 (human double minute 2) agents target HDM2, stabilize p53, and reactivate the p53 signaling pathway to kill cancer cells [27,162,163,164]. Nutlin-3a is one such chemical repressor; by binding HDM2, nutlin 3a reduces HDM2 function, thus stabilizing p53, and has been found to be efficacious in both in vivo and in vitro models, including breast cancer. It is currently undergoing phase III clinical trials [162,163,164,165].

**Figure 4:**
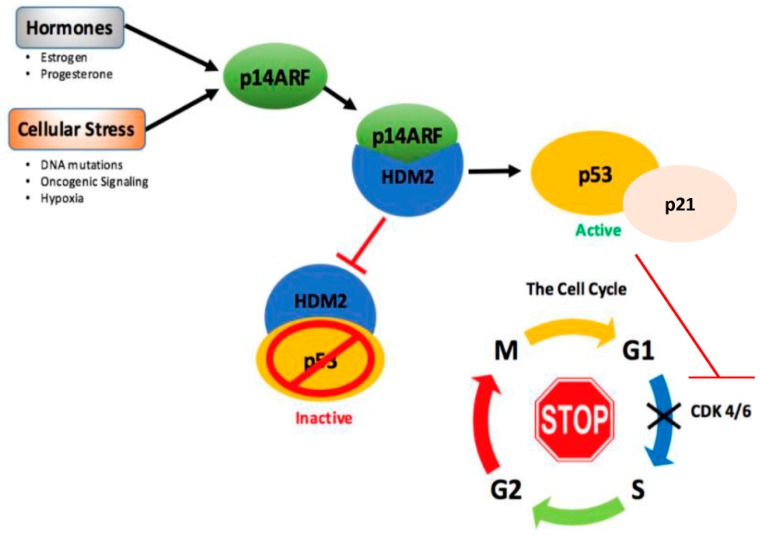
The p14ARF-p53 pathway. HDM2 sustains low basal levels of p53 by its continuous degradation [160,161]. p14ARF, activated by cellular stress signals and potentially regulated by estrogen and progesterone hormones in the breast [93,168,169,170,171], causes inhibition of the HDM2–p53 complex, therefore stabilizing p53. p53 is able to bring about cell cycle arrest at the G1/S phase through the activation of the CDK inhibitor p21, which inhibits downstream CDK 4 and 6 [66,68,69]. Both CDK 4 and 6 are well-known mediators of G1/S cell cycle progression, hence their inhibition by p21 halts the cell cycle [66].

p53 is commonly silenced in breast cancer by loss of upstream/downstream mechanisms. A key natural, endogenous regulator of p53 is the tumor suppressor p14 alternative reading frame (p14ARF). The p14ARF protein is an upstream positive regulator of p53 through its binding to HDM2 and preventing p53 degradation, thus stabilizing p53 expression and activating p53 function [166,167,168] (Figure 4). 

p14ARF is activated in response to adverse environmental stimuli to prevent hyperproliferation of cells to prevent cancer. The upstream p53 regulator, p14ARF, is silenced by methylation, deletion, or mutation in many breast cancers [166,172,173]. As such, therapeutic interest in re-activating the p14ARF-p53 pathway via re-introducing p14ARF or utilizing p14ARF mimetics to induce p53 (such as Nutlin-3a) has gained momentum. Early studies into re-activating the p14ARF-p53 pathway within breast cancer cells revealed rapid induction of apoptosis. However, these models utilized an adenoviral vector system expressing high, non-physiologically relevant levels of p53 [174]. Our laboratory has developed an inducible vector system to express controlled, physiologically relevant levels of p14ARF to activate the p53 signaling pathway in hormone-dependent breast cancer cells [59,93]. These studies demonstrated that p14ARF induces the p53-p21-retinoblastoma (Rb) pathway to induce cell cycle arrest; however, the cells did not undergo apoptosis, but remained metabolically active and viable [59,93]. Our studies linked the activation of p14ARF-p53 signaling directly to changes in breast cancer cellular metabolism. 

### 4.2. p53 Regulates Mitochondria Dynamics in Breast Cancer

Although mitochondrial dynamics has been observed to be influenced by p53 (Section 3.4), there is very little understanding of this process in breast cancer. In breast cancer, as in most cancers, p53 is reactivated by chemotherapy or radiation therapy to induce mitochondria-mediated cell death or permanent cell cycle arrest (Section 2.1). In our studies, in the absence of external stress factors, p14ARF re-activation of the p53 pathway rapidly induced cell cycle arrest (within 6 h) in breast cancer cells. Instead of the expected classical p53–mitochondria-induced apoptosis, p53 promoted major metabolic reprogramming in hormone-dependent breast cancer cells in favor of increased cellular function and viability [59,93]. Mitochondrial biomass, membrane potentiality, and mitochondria activity were enhanced, as well as metabolic and cellular morphological changes [59,93]. Therefore, p53 may be regarded as a pendulum between mitochondrial survival on one hand and mitochondrial-induced cell death on the other, depending on the cellular environment. Reactivation of the p14ARF-p53 pathway in ER+ breast cancer cells did not overtly alter the p53-dependent annexin A5 apoptotic marker associated with mitochondria-mediated cell death [59,93,94]. However, expression of other annexin family members and associated proteins, which control calcium flux, were enhanced [59,93,94]. Our findings supported the role of p53 as a key orchestrator of annexin/S100A regulation. In this study, annexins A1, A2, A4, A6, and A9 were upregulated downstream of the p53 signaling pathway [94]. Although the consequences of p53 differential regulation of the annexins/S100A family is not clear, intracellular calcium homeostasis and regulation of mitochondria function are not mutually exclusive [175]. For example, A6 has been reported to be an important protein in the regulation of mitochondrial morphogenesis, fission, and fusion, and loss of A6 expression is associated with fragmented mitochondria and impaired membrane potentiality and impaired oxidative respiration [176]. Regulation of calcium signaling is essential for mammary gland function and deregulation of calcium homeostasis is associated with cancer pathophysiology. 

This p53-attenuated apoptosis aligns nicely with a series of studies, illustrating that the estrogen receptor (ERα) interacts with p53, leading to an intrusion in p53 downstream signaling targets [177,178,179]. ERα signaling pathways and its expression within cancer and normal breast physiology have been extensively documented [180,181,182]; however, the interaction between ERα and p53 is novel within breast cancer. Konduri and colleagues first observed that p53 and ERα interact directly, leading to the downregulation of p53 alongside its downstream signaling targets such as p21 [178]. Further investigations by Brown and colleagues confirmed this interaction and showed that repression of p53 by ERα led to a lack of repression of genes involved in resisting apoptosis, thus hindering the ability of p53 to prompt apoptosis [177]. The data suggest a mechanism of resistance to treatments mediated through ERα, whereby functionally active p53 can become repressed in ER+ breast cancers. This also begs the question, does p53 interaction with ER+ in normal breast physiology to prevent cell death and divert the course of metabolic function?

### 4.3. Novel Avenues for Targeting p53 in Drug Resistance, Recurrence, and Metastasis in Breast Cancer

Increasing evidence supports the notion that dysregulated metabolism confers drug resistance and recurrence in breast cancer [156]. Breast cancer cells, like most cancer cells, use glucose and glutamine to survive, so, conceptually, targeting the energy-related metabolic pathways for therapeutic intervention in breast cancer is an exciting idea [183]. However, there is a fine balance between p53 regulation of mitochondrial processes, cell death, or cell survival in normal and cancer tissues. Therefore, targeting metabolism to prevent breast cancer recurrence has inherent problems: (1) All normal and cancer cells utilize both glycolysis and oxidative respiration for energy requirements; (2) different sub-types of breast cancer have different metabolic requirements and p53 status; (3) the complexities associated with the constant changes in breast metabolism and the emerging cross-talk of p53 with hormone receptors and other signaling pathways. This requires a more in-depth understanding of the intricacies of p53’s influence on metabolism in normal and breast cancer cells. 

An interesting advancement in our understanding of maladaptive metabolism and cancer came from studies in exercise physiology. Exercise, which alters cellular metabolism, has long been known to reduce breast cancer risk [184]. Aerobic exercise has long been known to increase mitochondria function and lactate clearance, and as well as to decrease glycolysis [185]. Lactate, once considered a waste product of anaerobic metabolism, is now regarded as an important fuel for cancer cell development and metastasis [186]. In addition, aerobic exercise has been shown to increase fat oxidation [187] As demonstrated in skeletal muscle, during exercise, p53 is a key molecule in substrate metabolic regulation and exercise-induced mitochondrial biogenesis, thus increasing mitochondrial respiratory activity [188]. As mentioned in Section 3.4, p53 regulates alterations in fission and fusion proteins, maintaining mitochondria morphology and respiratory capacity in muscle [58]. Perhaps a clearer understanding of the effects of exercise on p53-cellular metabolic reprogramming may provide clues for the prevention of breast cancer and new avenues for breast cancer treatment options.

## 5. Conclusions

The studies highlighted in this review reveal that p53 has a plethora of functions in normal and cancer metabolism beyond the classical mitochondrial death syndrome. Our major research focus is on understanding hormone-dependent cancer origin, treatment resistance, and recurrence in breast cancer. It is important to understand the role of p53 in the regulation of cancer metabolism as well as its underlying mechanism and how it applies directly to breast cancer. The mitochondrial targets of p53 that are unfolding would provide a platform to further investigate the role of p53 in modulating mitochondrial metabolic pathways. Future studies would include confirming p53 interactions with the aforementioned mitochondrial proteins, along with formulating a cohesive timeline for the changing metabolic phenotypes associated with p53’s evolving role as a master metabolic regulator in normal mammary cells and breast cancer cell evolution. 

The ultimate goal will be to elucidate the metabolic functions of p53 within breast cancer metabolism, which will reveal critical metabolic hotspots that cancers advantageously re-engineered for sustenance. Full understanding of these events would pave the way for the development of novel therapeutics targeting breast cancer metabolism and eventually new strategies to fight breast cancer.

## Figures and Tables

**Table 1:** Major breast cancer receptor sub-types and standard therapies.

Receptor Status [21,31]	Prevalence (%)	Endogenous Ligand	Standard Therapy Type
ERα+/PR+	70–80%	Estradiol/Progesterone	Endocrine Therapy
HER2+	15–20%	EGF, TGF-α	HER2-targeted Therapy
TNBC	15%	-	Chemotherapy/Radiotherapy

Note: ER, PR, and HER2 receptors are listed alongside their associated prevalence in breast cancers. TNBC refers to an absence of all three receptor types; this tumor type is notoriously difficult to treat [32]. The typical endogenous binding ligands and treatment type are also shown with the respective receptor profile.
